# Differentiating PSP from MSA using MR planimetric measurements: a systematic review and meta-analysis

**DOI:** 10.1007/s00702-021-02362-8

**Published:** 2021-06-08

**Authors:** Beatrice Heim, Florian Krismer, Klaus Seppi

**Affiliations:** Department of Neurology, Medical University of Innsbruck, Anichstrasse 35, 6020 Innsbruck, Austria

**Keywords:** Imaging biomarker, Cerebral pedunculi, Cerebellar pedunculi, Meta-analysis, Midbrain, Pons-to-midbrain ratio, Magnetic Resonance Parkinson Index, Progressive supranuclear palsy, Anatomical likelihood estimation, Seed-based D mapping

## Abstract

Differential diagnosis of parkinsonian syndromes is considered one of the most challenging in neurology. Quantitative MR planimetric measurements were reported to discriminate between progressive supranuclear palsy (PSP) and non-PSP-parkinsonism. Several studies have used midbrain to pons ratio (*M*/*P*) and the Magnetic Resonance Parkinsonism Index (MRPI) in distinguishing PSP patients from those with Parkinson's disease. The current meta-analysis aimed to compare the performance of these measures in discriminating PSP from multiple system atrophy (MSA). A systematic MEDLINE review identified 59 out of 2984 studies allowing a calculation of sensitivity and specificity using the MRPI or *M*/*P*. Meta-analyses of results were carried out using random effects modelling. To assess study quality and risk of bias, the QUADAS-2 tool was used. Eight studies were suitable for analysis. The meta‐analysis showed a pooled sensitivity and specificity for the MRPI of PSP versus MSA of 79.2% (95% CI 72.7–84.4%) and 91.2% (95% CI 79.5–96.5%), and 84.1% (95% CI 77.2–89.2%) and 89.2% (95% CI 81.8–93.8%), respectively, for the *M*/*P*. The QUADAS-2 toolbox revealed a high risk of bias regarding the methodological quality of patient selection and index test, as all patients were seen in a specialized outpatient department without avoiding case control design and no predefined threshold was given regarding MRPI or *M*/*P* cut-offs. Planimetric brainstem measurements, in special the MRPI and *M*/*P*, yield high diagnostic accuracy for the discrimination of PSP from MSA. However, there is an urgent need for well-designed, prospective validation studies to ameliorate the concerns regarding the risk of bias.

**Supplementary Information:**

The online version contains supplementary material available at 10.1007/s00702-021-02362-8.

## Introduction

Differential diagnosis of neurodegenerative Parkinsonian syndromes, including Parkinson's disease (PD), progressive supranuclear palsy (PSP), and multiple system atrophy (MSA), in early disease stages when clinical signs are subtle is considered as one of the most challenging in neurology.

PSP is an adult-onset progressive neurodegenerative disorder leading to supranuclear vertical gaze palsy, postural instability with falls, bradykinesia, and axial rigidity (Williams et al. 2005). There are various phenotypes of PSP, such as PSP-Richardson’s syndrome (PSP-RS), the parkinsonian variant of PSP (PSP-parkinsonism, PSP-P), PSP with predominant corticobasal syndrome (PSP-CBS), PSP with behavioral variant frontotemporal dementia (PSP-F), and PSP with progressive non-fluent aphasia (PSP-PNFA) (Höglinger et al. 2017). Atrophy of midbrain and superior cerebellar peduncle (SCP) are associated with PSP, and atrophy of pons and middle cerebellar peduncle (MCP) with the Parkinson variant of multiple system atrophy (MSA‐P), respectively (Nicoletti et al. 2006; Paviour et al. 2005). The midbrain‐to‐pontine area ratio (*M*/*P*) and the MR parkinsonism index (MRPI) were introduced because single measurement of these brain structures failed to differentiate neurodegenerative parkinsonian syndromes on an individual basis (Paviour et al. 2005; Seppi and Poewe 2010) and these quantitative MR planimetric measurements have been reported to differentiate PSP from PD and MSA with high diagnostic accuracy (Heim et al. 2018, 2021; Mangesius et al. 2018). With regard to future therapeutic approaches, new studies are planned to influence the course of neurodegenerative diseases and, therefore, early diagnostic accuracy is crucial. A recent meta-analysis showed a high performance of the MRPI and *M*/*P* in differentiating patients with PSP from patients with PD with a pooled sensitivity and specificity of 98% and 99% as well as 92% and 94%, respectively (Zhang et al. 2019).

The purpose of this meta-analysis was to detect diagnostic accuracy of MRPI and *M*/*P* in discriminating between PSP from MSA.

## Methods

### Search strategies and study selection

Two raters (BH, KS) systematically searched the electronic MEDLINE database PubMed by two combination of terms as “magnetic resonance imaging” or “magnetic resonance parkinson* index” and “progressive supranuclear palsy” or “Parkinson* disease” “magnetic resonance parkinsonism index” + “parkinson disease” or “progressive supranuclear palsy” or “multiple system atrophy” with time limit from 1 January 2005 to 20 November 2020. The two raters searched various alterations in spelling due to the pronounced heterogeneity. As the first planimetric MRI study using the *M*/*P* or the MRPI was published in 2005 (Oba et al. 2005), we defined a time limit starting in 2005. The final search was conducted on the 20th of November 2020 and resulted in a total of 2984 articles. The detailed search strategies are given in Supplementary Table 1.

For this meta-analysis, we included MRI studies using MRPI and *M*/*P* area ratio or both to distinguish PSP from MSA patients. For further analysis, papers had to satisfy the following predefined eligibility criteria: (1) papers were required to be published in English language; (2) both PSP and MSA patients were included in the study; (3) studies reported either true positive, true negative, false positive, and false negative rates, or overall sample size and sensitivity and specificity values. Our meta-analysis complied with the Preferred Reporting Items for Systematic Reviews and Meta-Analyses (PRISMA) statement (Moher et al. 2009).

Exclusion criteria were: (1) studies including only one PSP or MSA patients without a control group; (2) review articles reporting no original data; (3) articles not giving either true positive, true negative, false positive, and false negative rates, or sensitivity and specificity values.

### Quality assessment

The Quality Assessment Tool for Diagnostic Accuracy Studies 2 (QUADAS-2) (Whiting et al. 2011), evaluated by Review Manager 5.3 (Nordic Cochrane Centre, Copenhagen, Denmark), was used to assess each study's methodological quality regarding risk of bias and concerns regarding applicability. Quality assessment was performed by two independent raters (BH, FK) and discordant ratings were resolved in a discussion of the two raters. Data extraction was done for each paper by the two independent investigators.

### Data analysis

This meta-analysis was carried out using the MRPI and *M*/*P* ratio to distinguish between PSP from MSA in early disease stages.

For statistical analysis, the following data were extracted from each of the studies: (1) number of participants in each group; (2) sensitivity and specificity, or alternatively, true positive, true negative, false positive, and false negative rates.

A bivariate model specified as a linear mixed model with known variances of the random effects implemented in the R package mada was applied to estimate overall sensitivity and the overall false positive rate (Reitsma et al. 2005). Chi-squared tests were applied to assess heterogeneity of sensitivities and specificities, the null hypothesis being in both cases, that all studies are equal.

The sensitivities and specificities of each study were summarized using the hierarchical summary receiver operating characteristics (HSROC) curve approach (Rutter and Gatsonis 2001) with 95% confidence intervals (95% CI). Moreover, the corresponding positive and negative likelihood ratios (LR) as well as diagnostic odds ratio (DOR) were estimated.

Between‐study heterogeneity was assessed using the I^2^ statistic, which provides a measure of the degree of inconsistency across studies describing the percentage of total variation attributable to heterogeneity rather than chance. I^2^ values up to 30% to 40% are considered as low heterogeneity, values up to 50% to 60% as moderate heterogeneity (http://handbook.cochrane.org/chapter_9/9_5_2_identifying_and_measuring_heterogeneity.htm).

## Results

### Study characteristics

**Fig. 1:**
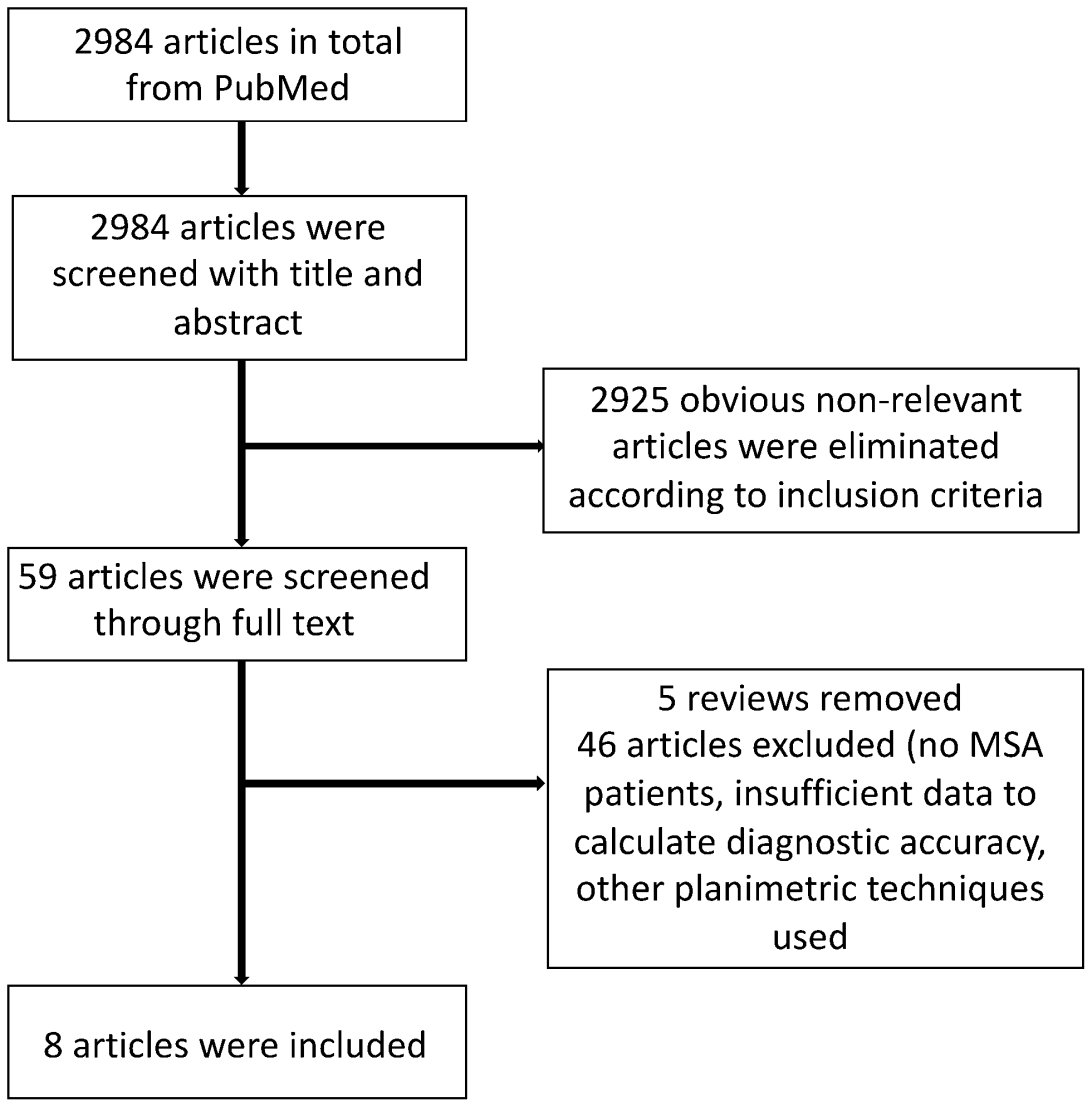
Flowchart for the identification of eligible studies

A total of 2984 papers were identified by the initial literature research. After review of the abstracts, 59 publications were selected for further review of the full texts. Only eight studies satisfied the predefined criteria as stated above and were deemed relevant for MRPI and/or *M*/*P* assessment. A detailed flow chart of the review process is shown in Fig. 1.

Two studies used 3 T field strength (Archer et al. 2020; Sakamoto et al. 2020), other three studies used 1.5 T field strength (Mangesius et al. 2018; Quattrone et al. 2008; Oba et al. 2005), and three studies used both 3 T and 1.5 T scanners (Constantinides et al. 2018; Sjöström et al. 2020; Möller et al. 2017).

All studies used established diagnostic criteria as a reference standard. All but one (Sakamoto et al. 2020) of the included studies used clinical criteria proposed by the National Institute of Neurological Disorders and Stroke and Society for PSP (NINDS‐SPSP) (Litvan et al. 1996). One of these studies (Archer et al. 2020) applied the recently revised MDS criteria for PSP (Höglinger et al. 2017) retrospectively to discriminate PSP patients into the two most common clinical predominance types of PSP (PSP-RS and PSP-P). One study used the MDS criteria for PSP diagnosis (Sakamoto et al. 2020) and included both PSP-RS and PSP-P patients but did not provide clinical data of the PSP patients. Two of the studies using the NINDS‐SPSP clinical criteria for PSP (Archer et al. 2020; Mangesius et al. 2018) included PSP-RS and PSP-P patients, the five other studies (Oba et al. 2005; Constantinides et al. 2018; Sjöström et al. 2020; Möller et al. 2017; Quattrone et al. 2008) included PSP-RS patients only. Four studies (Quattrone et al. 2008; Constantinides et al. 2018; Möller et al. 2017; Oba et al. 2005) included only probable MSA, whereas three studies (Archer et al. 2020; Mangesius et al. 2018; Sakamoto et al. 2020) did not define clinical disease category of MSA (possible vs. probable). Two studies (Möller et al. 2017; Sjöström et al. 2020) included patients with MSA of the cerebellar type (MSA-C) as well, but only MSA patients of the parkinsonian type were included in this meta-analysis. Five out of eight studies (Constantinides et al. 2018; Sjöström et al. 2020; Quattrone et al. 2008; Mangesius et al. 2018; Sakamoto et al. 2020) reported consecutive patient recruitment, and four (Möller et al. 2017; Archer et al. 2020; Quattrone et al. 2008; Mangesius et al. 2018) reported blinded procedure (Table 1).

**Table 1:** Overview of studies included in the analysis with demographic details

	PSP				MSA			
	*n*	*m*/*f*	Disease duration (years)	H&Y	*n*	*m*/*f*	Disease duration (years)	H&Y
Archer et al. (2020)	16^a^	7/9	3.0 ± 2.7	3.06 ± 1.18	17	12/5	4.6 ± 3.0	3.35 ± 1.00
Constantinides et al. (2018)	24	13/11	3.3 ± 1.8	n.a	9	7/2	3.4 ± 2.8	n.a
Mangesius et al. (2018) (Cohort 1)	55^b^	28/27	3.7 ± 1.6	3.51 ± 0.88	63	28/35	4.1 ± 2.4	3.42 ± 1.01
Mangesius et al. (2018) (Cohort 2)	17^c^	10/7	0.9 ± 0.4	2.44 ± 0.50	12	7/5	0.9 ± 0.4	2.13 ± 0.64
Mangesius et al. (2018) (Cohort 3)	23^d^	16/7	2.1 ± 1.5	3.14 ± 0.74	22	12/10	2.1 ± 1.5	3.39 ± 0.596
Möller et al. (2017)	106	60/46	3.2 ± 0.2	n.a	60	38/22	3.6 ± 0.3	n.a
Oba et al. (2005)	21	13/8	2.8 ± 1.3	n.a	25	6/19	7.8 ± 3.8	n.a
Quattrone et al. (2008)	33	23/10	3.0 ± 1.6	4 (2.5–5)	19	5/14	4.6 ± 3.1	3 (1.5–5)
Sakamoto et al. (2020)	20^e^	10/10	n.a	n.a	8	n.a	n.a	n.a
Sjöström et al. (2020)	29	18/11	3.1 ± 1.8	3.4 ± 0.9	27 (3 MSA-C)^f^	13/14	2.4 ± 1.5	3.4 ± 1.1

^a^11 had PSP-RS and 5 PSP-P

^b^33 had PSP-RS and 12 PSP-P

^c^12 had PSP-RS and 5 PSP-P

^d^15 had PSP-RS and 8 PSP-P

^e^15 had PSP-RS and 5 PSP-P (information not provided in the paper, according to Table 2 of the paper it seems that 15 patients had PSP-RS and 5 PSP-P)

^f^MSA-C patients were excluded for the meta-analysis

### Meta-analysis

**Fig. 2:**
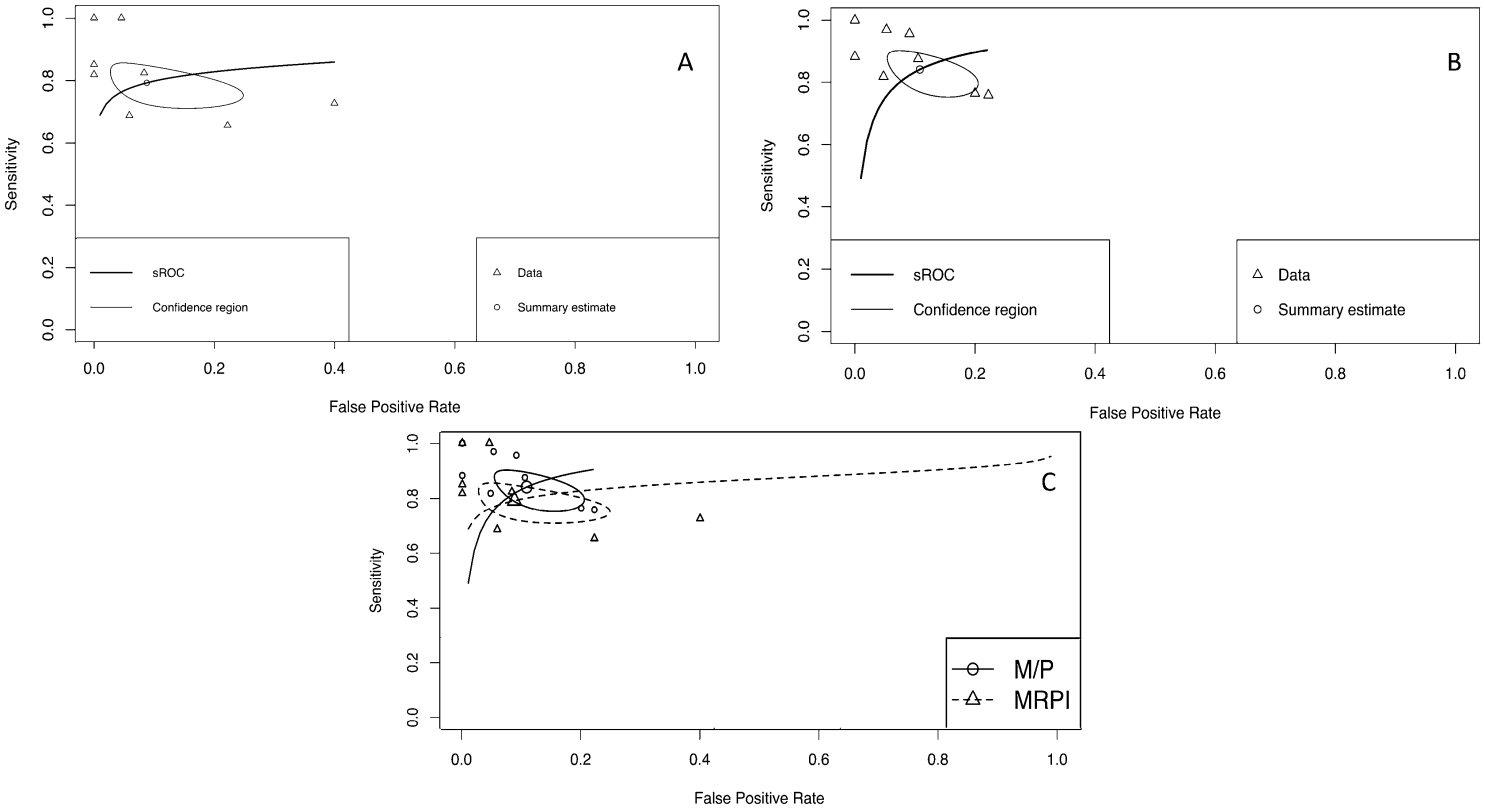
Summary receiver operating characteristic curve of MRPI (**A**) and *M*/*P* (**B**) analysis and comparison of MRPI vs. *M*/*P* (**C**). Area under the curve represents accuracy of diagnosis

The meta‐analysis showed an overall pooled sensitivity and specificity for the MRPI of PSP versus MSA of 79.2% (95% CI 72.7‐84.4%) and 91.2% (95% CI 79.5–96.5%) and 84.1% (95% CI 77.2–89.2%), respectively (Table 2), with a positive LR of 10.2 (median 9.00, 95% CI 3.690–23.7), a negative LR of 0.234 (median 0.23; 95% CI 0.165–0.326), and a DOR of 47.800 (median 39.50; 95% CI 11.800–132.00). The pooled sensitivity and specificity were 84.1% (95% CI 77.2–89.2%) and 89.2% (95% CI 81.8–93.8%), respectively, for the *M*/*P* (Table 2) with a positive LR of 8.13 (median 7.76, 95% CI 4.390–14.00), a negative LR of 0.183 (median 0.179; 95% CI 0.118–0.269), and a DOR of 48.700 (median 43.50; 95% CI 17.200–110.00). Pooled sensitivity and specificity values for MRPI and *M*/*P* ratio are demonstrated in Figs. 2A–C.

**Table 2:** Overview of blinded studies included in the analysis assessing MRPI and *M*/*P*

	MRPI			*M*/*P*		
	Cut-off	Sensitivity	Specificity	Cut-off	Sensitivity	Specificity
Archer et al. (2020)	n.a	0.69	0.94	n.a	n.a	n.a
Constantinides et al. (2018)	> 12.6	1.00	1.00	≤ 0.22	0.88	0.89
Mangesius et al. (2018) (Cohort 1)	> 15.62	0.82	1.00	< 0.18	0.82	0.95
Mangesius et al. (2018) (Cohort 2)	> 15.62	0.82	0.92	< 0.18	0.88	1.00
Mangesius et al. (2018) (Cohort 3)	> 15.62	1.00	0.95	< 0.18	0.96	0.91
Möller et al. (2017)^a^	8.51	0.73	0.60	0.22	0.76	0.80
Oba et al. (2005)	n.a	n.a	n.a	0.15	1.00	1.00
Quattrone et al. (2008)	≥ 12.85	1.00	1.00	≤ 0.22^b^	0.97	0.95
Sakamoto et al. (2020)	> 11.60	0.85	1.00	n.a	n.a	n.a
Sjöström et al. (2020)^a^	> 15.38	0.66	0.78	< 0.42	0.76	0.78

^a^MSA-C patients were excluded for the meta-analysis

^b^In the paper the ratio of pontine/midbrain area is given (i.e. 4.62)

There was low between-study heterogeneity as suggested by *I*^2^ score of 12.8% for the MRPI, respectively. For the *M*/*P* ratio, between-study heterogeneity was 6.5%.

### Quality assessment (Fig. 3A, B)

**Fig. 3:**
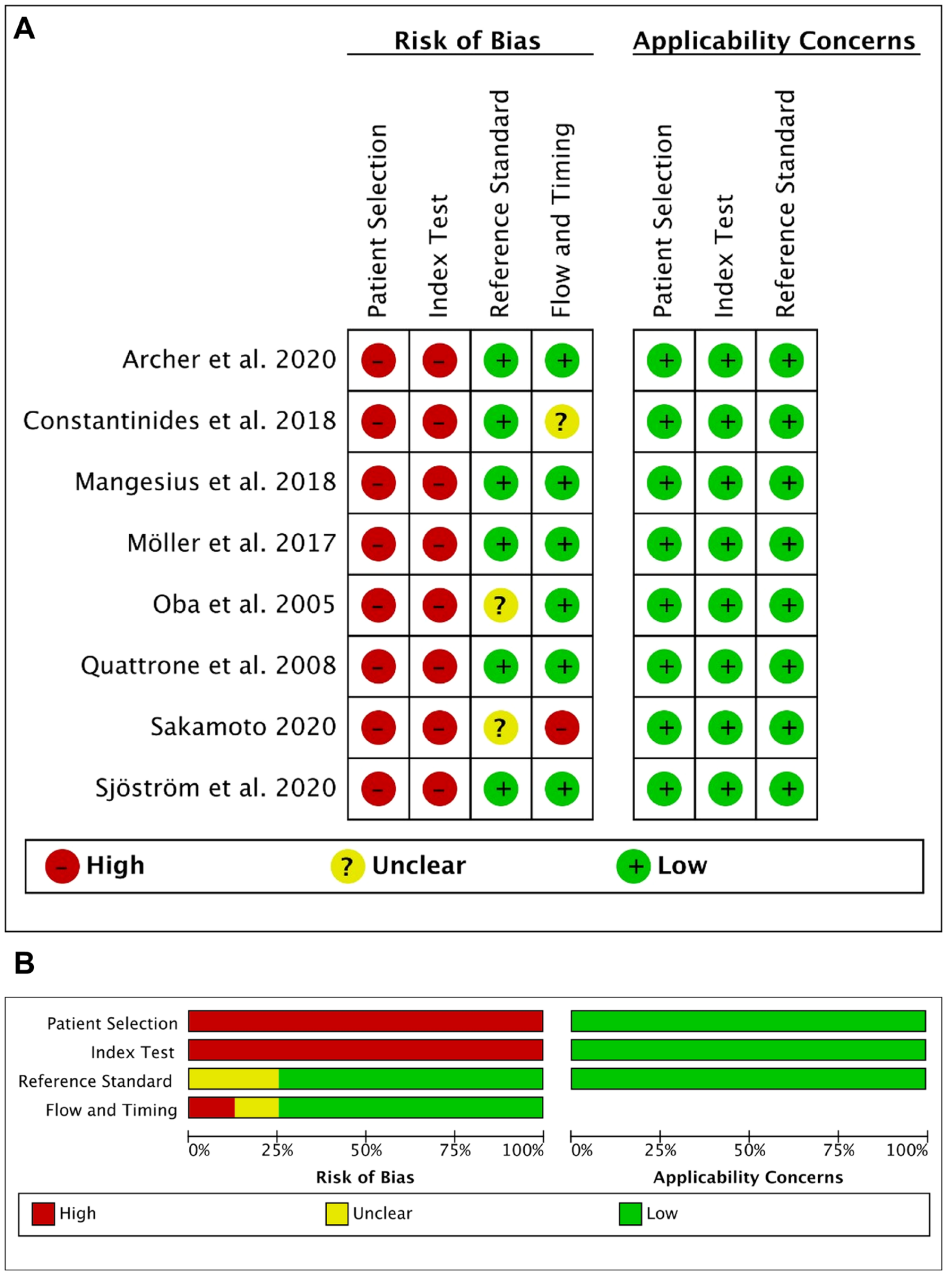
Methodological analysis of the included studies based on QUADAS-2 assessment

The accuracy of MRPI vs. *M*/*P* ratio to distinguish between PSP and MSA was examined in seven publications. All studies showed a high risk of bias regarding the methodological quality of patient selection and index test, as all patients were seen in specialized outpatient departments without avoiding case control design and no predefined threshold was given regarding MRPI or *M*/*P* cut-offs.

**Fig. 4:**
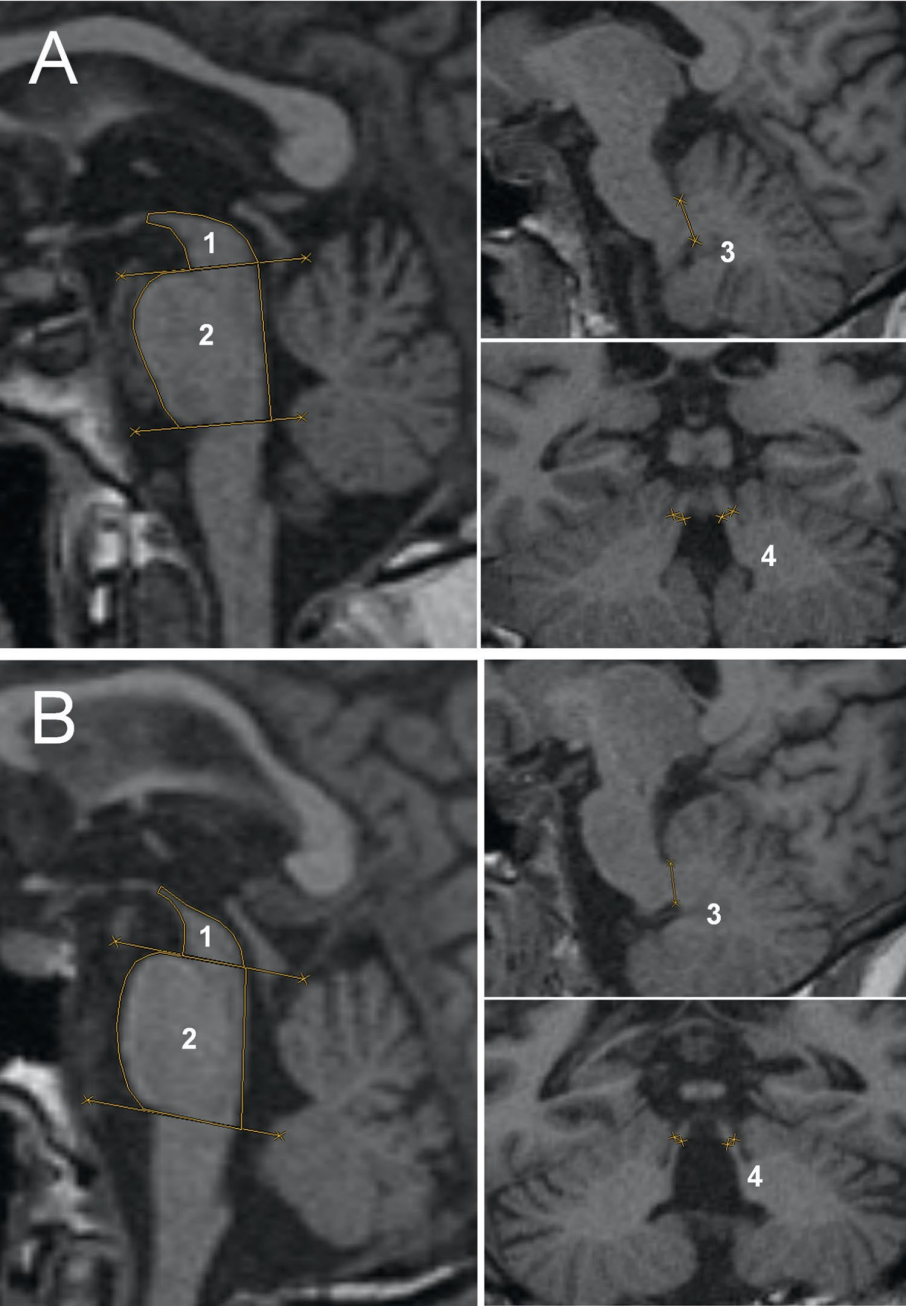
Adapted and reprinted with permission from Mangesius et al. (2018): performance of MR planimetric measurements in a **A** PD and a **B** PSP patient: **1** midbrain area, **2** pons area, **3** MCP diameter, and **4** SCP diameter

Criterion-related and construct validity was assessed by comparing different MRI planimetric measurements as comparator with diagnostic criteria in all included studies (Fig. 4).

## Discussion

In this meta-analysis, we aimed to evaluate the diagnostic accuracy of planimetric measurements, i.e. MRPI and *M*/*P*, for differential diagnosis of PSP versus MSA. However, MR planimetric measurements seem to provide an early possibility to detect specific atrophy patterns in different neurodegenerative parkinsonian disorders. The quantitative synthesis of the present systematic review showed an overall sensitivity and specificity of the MRPI for the differential diagnosis of PSP versus MSA of 79.2% (95% CI 72.7–84.4%) and 91.2% (95% CI 79.5–96.5%), respectively, with a positive LR of 10.2 (median 9.00, 95% CI 3.690–23.7), a negative LR of 0.234 (median 0.23; 95% CI 0.165–0.326), and a DOR of 47.800 (median 39.50; 95% CI 11.800–132.00). For the *M*/*P*, overall sensitivity and specificity reached 84.1% (95% CI 77.2–89.2%) and 89.2% (95% CI 81.8–93.8%), respectively, with a positive LR of 8.13 (median 7.76, 95% CI 4.390–14.00), a negative LR of 0.183 (median 0.179; 95% CI 0.118–0.269), and a DOR of 48.700 (median 43.50; 95% CI 17.200–110.00).

In detail, all of the included studies (Oba et al. 2005; Archer et al. 2020; Mangesius et al. 2018; Möller et al. 2017; Sjöström et al. 2020; Sakamoto et al. 2020; Quattrone et al. 2008; Constantinides et al. 2018) assessed the MRPI and *M*/*P* in clinically diagnosed PSP or MSA, which likely overestimates the sensitivity of the diagnostic test by excluding patients with suspected disease but an unconfirmed diagnosis (i.e. difficult-to-diagnose patients, QUADAS-2 signalling question for inappropriate exclusion (Whiting et al. 2011)). Interestingly, one study included a cohort of clinically uncertain parkinsonian syndromes (CUPs) (Mangesius et al. 2018), reporting a sensitivity of 82% and a specificity of 92% in discriminating PSP from MSA using the MRPI, and a sensitivity of 88% and a specificity of 100% using the *M*/*P* ratio.

Comparing these two MR planimetric methods, *M*/*P* provides numerically higher sensitivity rates to distinguish between PSP and MSA than the MRPI, whereas the MRPI is more beneficial in discriminating specificity and false positive rates.

Our meta-analysis showed higher between-study heterogeneity of the MRPI than of the *M*/*P* (12.8% vs. 6.5%). This is possibly explained by the easier and faster method of the *M*/*P* to apply than MRPI (Mangesius et al. 2018; Hussl et al. 2010). Nevertheless, using automated and observer-independent MRPI assessment approaches will improve interrater reliability and substantially reduce the time needed to perform the analyses.

Formal assessment of interrater variability was performed in three of the above mentioned studies (Oba et al. 2005; Sjöström et al. 2020; Möller et al. 2017): one study found limited interrater reliability for the cerebellar peduncles and, therefore, for the MRPI, and strong reliability for the pons and midbrain area (Möller et al. 2017). One study (Oba et al. 2005) found excellent intraobserver correlation for pons and midbrain area. Excellent interrater correlation for *M*/*P*, good for MRPI, and again excellent for the MRPI2.0 were found in another study (Sjöström et al. 2020). Inter-rater intraclass correlation (ICC) for the two raters was excellent for MP-ratio (0.94), good for MRPI 1.0 (0.86) and excellent for MRPI 2.0 (0.93).

All of the included studies (Oba et al. 2005; Sjöström et al. 2020; Archer et al. 2020; Constantinides et al. 2018; Möller et al. 2017; Quattrone et al. 2008; Mangesius et al. 2018; Sakamoto et al. 2020) exploited a test threshold tailored to the study sample in attempt to optimize sensitivity and/or specificity which may lead to overestimation of test performance. Test performance is likely to be poorer in an independent sample where a predefined threshold is used (Leeflang et al. 2008). However, when looking at the individual studies each, threshold measures were very similar.

To assess study quality and risk of bias, we used the QUADAS-2 tool. QUADAS-2 consists of four key domains that discuss patient selection, index test, reference standard, and flow of patients through the study and timing of the index tests and reference standard (flow and timing) (Whiting et al. 2011). Results indicate bias risk and applicability. All but one study used an acceptable reference standard independent of the index test and most studies had an appropriate flow and timing of index and reference test. However, QUADAS-2 signalling questions indicated risk of bias with regards to patient selection and the performance of the index test in all included studies. Patient selection of the studies may have overestimated diagnostic accuracy by either enrolling only consecutive participants with confirmed diagnoses or applying a case–control design. Latter is prone (similarly to the abovementioned design flaws) to exaggerate diagnostic accuracy (Lijmer et al. 1999; Whiting et al. 2004). Moreover, all but one study (Mangesius et al. 2018) used cut-off values of the index test established in the patient groups studied. Indeed, a prediction model showing acceptable or good performance based on internal validation in the development data set, will not necessarily behave similarly in a different group of individuals (Altman et al. 2009; Hendriksen et al. 2013). Ideally, the performance of a prediction model should be assessed with patient data not used in the development process of an index test (Hendriksen et al. 2013; Mangesius et al. 2018).

Three studies (Oba et al. 2005; Constantinides et al. 2018; Sjöström et al. 2020) did not report whether “blinding” was applied for interpretation of the index test—if a rater was aware of the results of the reference test, it would introduce subjectivity to interpreting index test results and again overestimate test performance.

The lack of post-mortem verification is another source of concern. Clinical misclassification cannot be excluded entirely, which could also impact test performance. While most studies were performed in PSP-RS patients, three studies also included PSP-P patients (Archer et al. 2020; Mangesius et al. 2018; Sakamoto et al. 2020), where diagnostic accuracy of both the MRPI and *M*/*P* might be lower compared to PSP-RS. Therefore, the MRPI2.0 and P/M2.0 including the measurement of the third ventricle width (MRPI or *M*/*P* multiplied by third ventricle width/frontal horns width ratio) were developed to increase diagnostic accuracy for PSP-P (Quattrone et al. 2018). Indeed, validation studies showed that these two measures were more powerful in discriminating PSP-P from PD than the MRPI and P/M (Heim et al. 2018, 2021; Quattrone et al. 2019), but there is a lack on studies exploring the diagnostic value of these two new measures in discriminating patients with PSP from MSA.

Although there is evidence that different scanner types do not influence brainstem-derived planimetric measurements, the studies used different scanner types and field strengths (1.5 T vs. 3 T), which might be a potential source for an increased variability (Mangesius et al. 2018).

In conclusion, brainstem-derived MR planimetric measures yield high diagnostic accuracy for the discrimination of PSP from MSA. However, there is an urgent need for well-designed, prospective blinded validation studies with predefined thresholds to ameliorate these concerns regarding the risk of bias.

## Supplementary Information

Below is the link to the electronic supplementary material.

Detailed search strategies (DOCX 15 kb)

Forest plot of sensitivity of MRPI for the diagnosis of PSP vs. MSA (JPG 1079 kb)

Forest plot of specificity of MRPI for the diagnosis of PSP vs. MSA (JPG 1058 kb)

Forest plot of sensitivity of M/P for the diagnosis of PSP vs. MSA (JPG 1027 kb)

Forest plot of specificity of M/P for the diagnosis of PSP vs. MSA (JPG 1593 kb)
