# Supplementary material for: Differentiating PSP from MSA using MR planimetric measurements: a systematic review and meta-analysis
Source: J Neural Transm (Vienna). 2021 Jun 8;128(10):1497–505. doi: 10.1007/s00702-021-02362-8 (PMC8528799; doi:10.1007/s00702-021-02362-8)
Supplement: Supplementary file 1 — Detailed search strategies (DOCX 15 kb) [file 702_2021_2362_MOESM1_ESM.docx]

Supplementary Table 1. Detailed search strategies.

| Databases | Search strategies | Search outcomes |
| --- | --- | --- |
| Pubmed | #1: ((ophthalmoplegia, progressive supranuclear[MeSH Terms]) AND (ophthalmoplegia, progressive supranuclear[MeSH Terms])) AND (palsy, progressive supranuclear[MeSH Terms]) | 2984 |
|  | #2: ((atrophy, multiple system[MeSH Terms]) AND (multiple system atrophies[MeSH Terms])) AND (multiple system atrophy syndrome[MeSH Terms]) |  |
|  | #3: (((parkinsonian diseases[MeSH Terms]) AND (parkinsonian disorders[MeSH Terms])) AND (parkinsonian syndrome[MeSH Terms])) AND (parkinsonian syndromes[MeSH Terms]) |  |
|  | #4: ((((idiopathic parkinson's disease[MeSH Terms]) AND (lewy body parkinson's disease[MeSH Terms])) AND (parkinson's disease[MeSH Terms])) AND (parkinson's disease, idiopathic[MeSH Terms])) AND (parkinson's disease, lewy body[MeSH Terms]) |  |
|  | #5: parkinsonism[Title/Abstract] |  |
|  | #6: #1 OR #2 OR #3 OR #4 OR #5 |  |
|  | #7: ((((((magnetic resonance imaging[Title/Abstract]) OR (planimetr*[Title/Abstract])) OR (magnetic resonance parkinson* index[Title/Abstract])) OR (MRPI[Title/Abstract])) OR (MRI[Title/Abstract])) OR (NMR Imaging[Title/Abstract])) OR (Spin Echo Imaging[Title/Abstract]) |  |
|  | #8: #6 AND #7 |  |
|  | #9: ("2005/01/01"[Date - Publication] : "2020/11/20"[Date - Publication]) |  |
|  | #10: #8 AND #9 |  |
|  | #11: english[Language] |  |
|  | #12: #9 AND #10 |  |
