# Supplementary figures and images for: Differentiating PSP from MSA using MR planimetric measurements: a systematic review and meta-analysis
Source: J Neural Transm (Vienna). 2021 Jun 8;128(10):1497–505. doi: 10.1007/s00702-021-02362-8 (PMC8528799; doi:10.1007/s00702-021-02362-8)

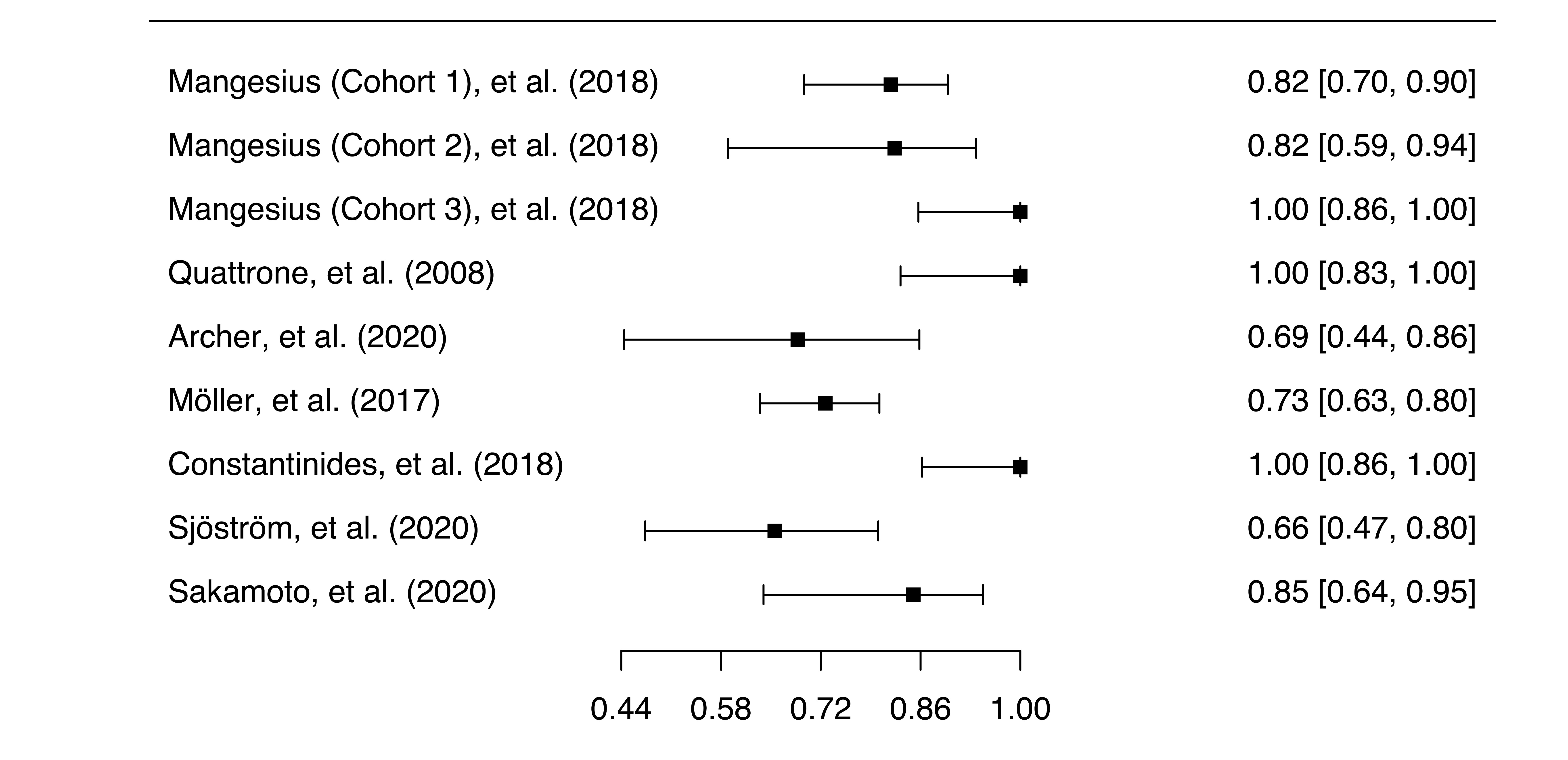

Supplement: Supplementary file 2 — Forest plot of sensitivity of MRPI for the diagnosis of PSP vs. MSA (JPG 1079 kb) [file 702_2021_2362_MOESM2_ESM.jpg]

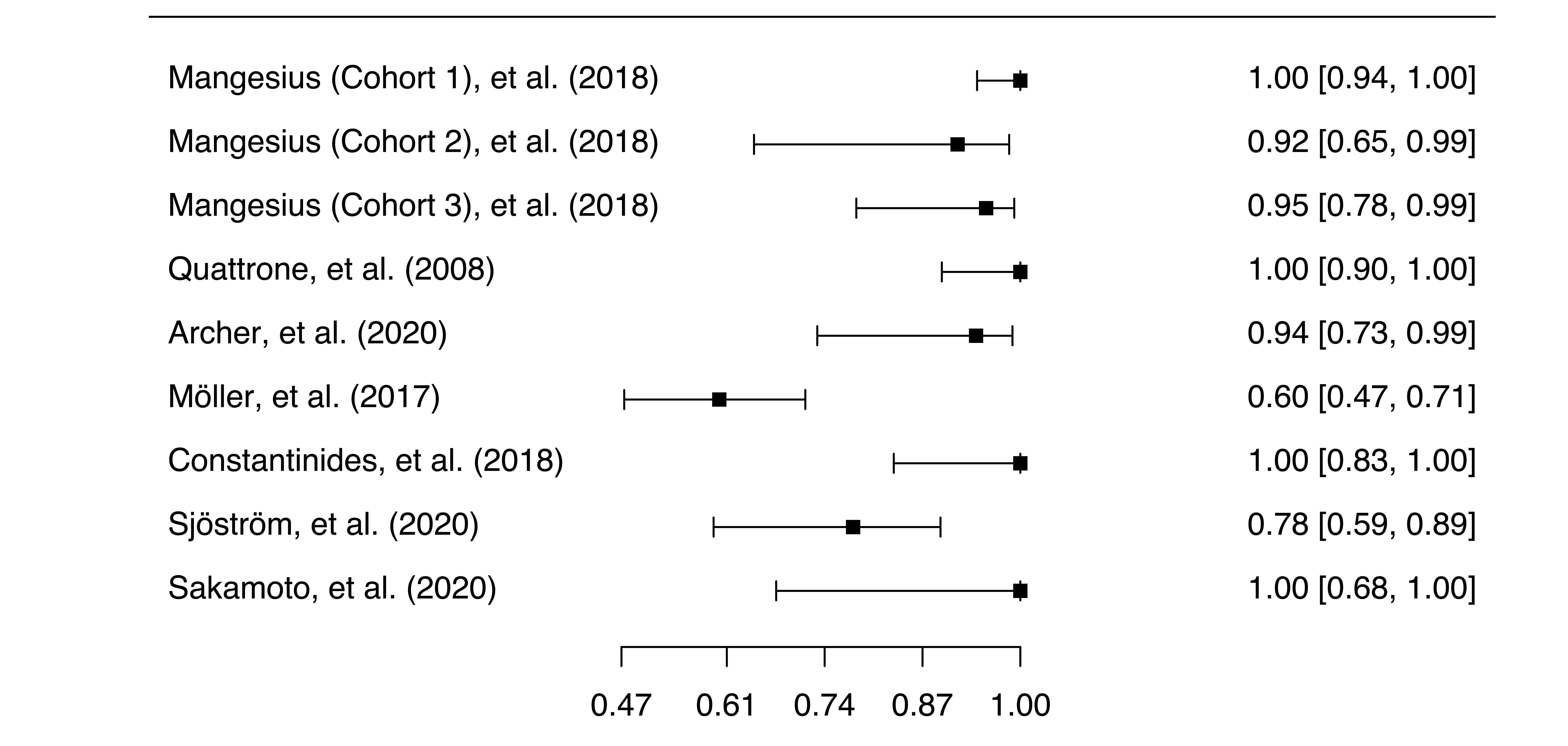

Supplement: Supplementary file 3 — Forest plot of specificity of MRPI for the diagnosis of PSP vs. MSA (JPG 1058 kb) [file 702_2021_2362_MOESM3_ESM.jpg]

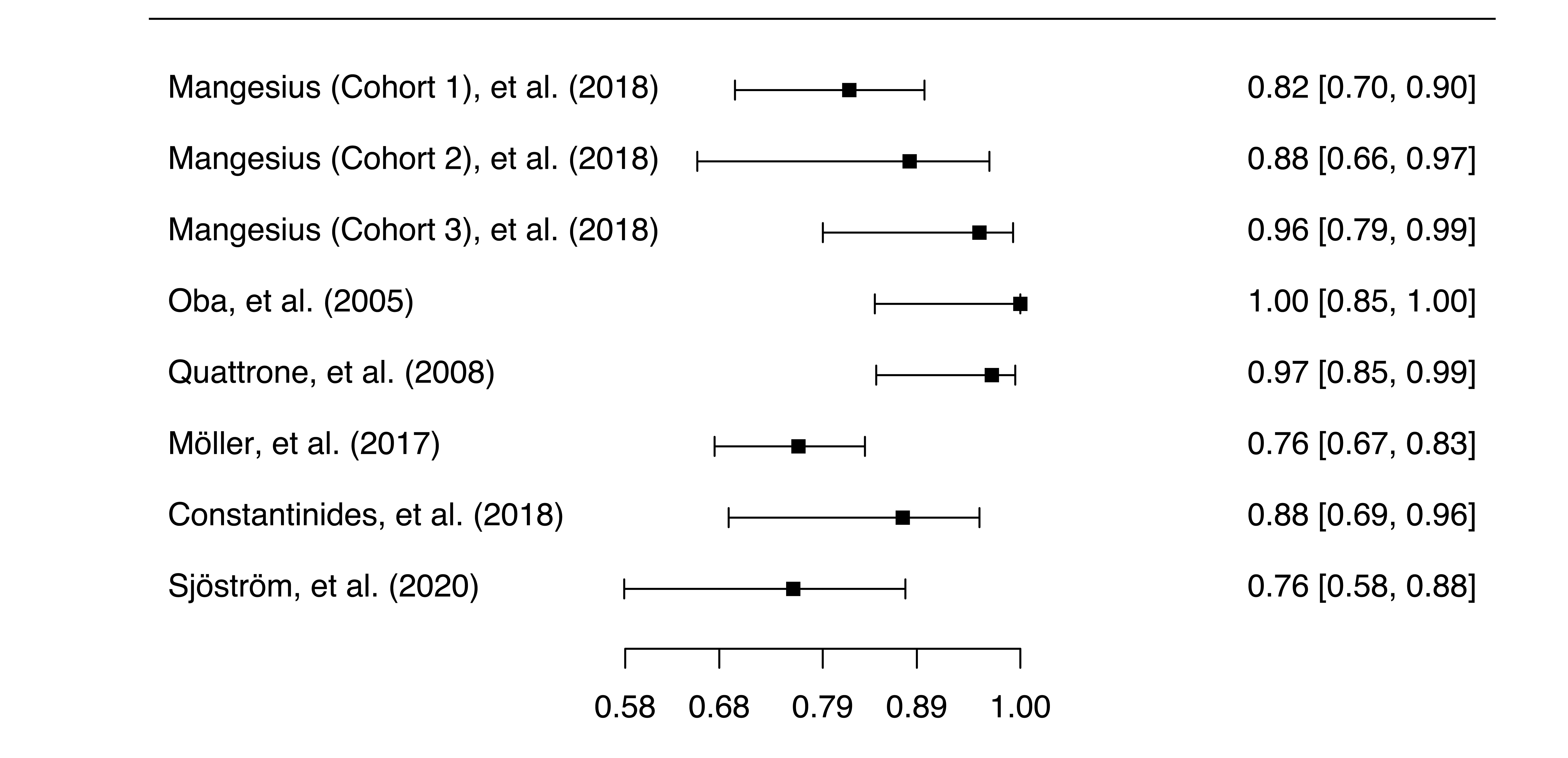

Supplement: Supplementary file 4 — Forest plot of sensitivity of M/P for the diagnosis of PSP vs. MSA (JPG 1027 kb) [file 702_2021_2362_MOESM4_ESM.jpg]

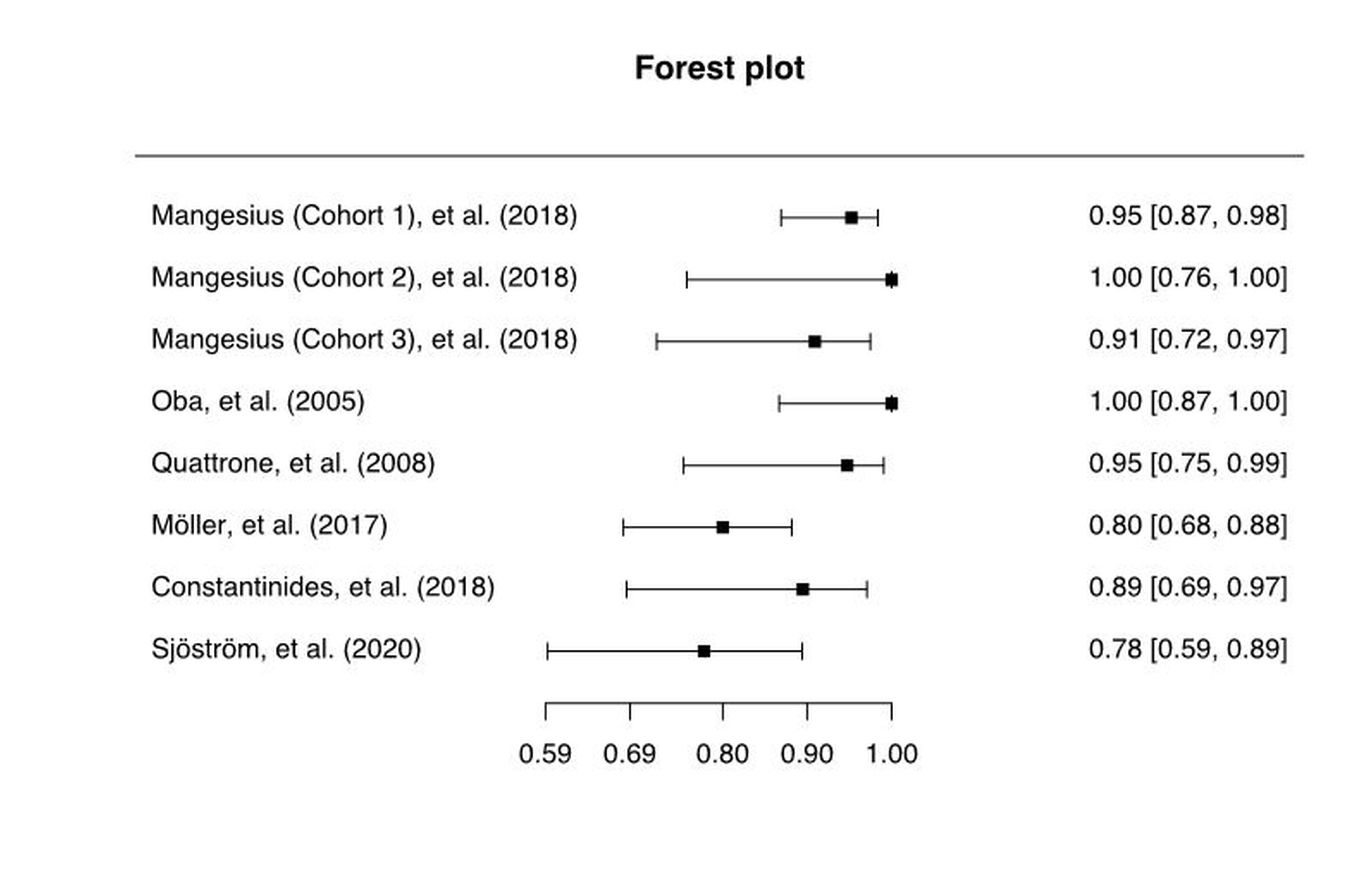

Supplement: Supplementary file 5 — Forest plot of specificity of M/P for the diagnosis of PSP vs. MSA (JPG 1593 kb) [file 702_2021_2362_MOESM5_ESM.jpg]
